# COVID-19 Pandemic, Determinants of Food Insecurity, and Household Mitigation Measures: A Case Study of Punjab, Pakistan

**DOI:** 10.3390/healthcare9060621

**Published:** 2021-05-22

**Authors:** Muhammad Aamir Shahzad, Ping Qing, Muhammad Rizwan, Amar Razzaq, Muhammad Faisal

**Affiliations:** 1 College of Economics and Management, Huazhong Agricultural University, Wuhan 430070, China; sivia.amir@gmail.com (M.A.S.); amar.razzaq@hotmail.com (A.R.); faisalgurmani@gmail.com (M.F.); 2 School of Economics and Management, Yangtze University, Jingzhou 434023, China; 3 Changjiang Belt Economic and Development Research Institute, Yangtze University, Jingzhou 434023, China

**Keywords:** COVID-19 pandemic, food insecurity, coping strategies, HFIAS, financial support

## Abstract

Policies centered to respond to the COVID-19 pandemic have created recessionary economic impacts. Adverse income shocks have caused malnutrition and food insecurity and have increased the need for food assistance. The present study examines the impact of the COVID-19 pandemic on food insecurity and investigates the determinants of food security and coping strategies in the Punjab province of Pakistan. Data were collected through the internet and received responses from 370 respondents. The household food insecurity access scale (HFIAS) model was applied to examine food insecurity, and a logit regression model was used to analyze its determining factors. The results illustrate that food insecurity substantially increased during the COVID-19 pandemic. Households’ demographics and socioeconomic factors have influence on food insecurity. Households with a large family size and people in quarantine found more food insecurity during the COVID-19 pandemic, while financial assistance played a role in a decline in food insecurity. Households handle the negative income shocks by eating less preferred food and getting support from government and charity organizations. It is suggested that stakeholders and responsible institutes provide financial assistance to support low-income families in order to enhance food security. Furthermore, policymakers should strengthen social safety nets and aid programs such as the Ehsas income program in the province of the country.

## 1. Introduction

The population of Pakistan is facing high levels of malnutrition and food insecurity [1]. One-fourth of the population is exposed to malnutrition and food insecurity, meaning they cannot fulfill basic intake requirements [2,3]. COVID-19 quarantine policies have led to unprecedented adverse effects on Pakistan’s already struggling food system, including the supply chain. Apart from the direct health effects of the COVID-19 pandemic, the policies responses, such as travel and trade restrictions, social distancing, and the closure of formal and informal indicators against COVID-19, have created declining economic impacts [4]. The food-insecure population has experienced adverse effects due to COVID-19 [5,6]. A reduction in small food outlet activity and a compromised open market have inhibited the ability to purchase food. On the one hand, disruptions in food systems lead to price increases; on the other hand, income loss results in a decline in the purchasing power, exaggerating the food and nutrition insecurity [5,6,7,8]. It is shown that people experiencing food insecurity have been affected more strongly by the far-reaching impact of COVID-19 in 2020, exposing multiple fragilities and vulnerabilities in the contemporary food system [9].

The literature about the impact of COVID-19 on food security is rare. Previous research has focused on virus policies responses to trade, transport, logistics, and other restrictions [9,10,11,12,13]. A couple of studies have focused on the interactions between health and economic policies [14,15]. In another study conducted by Kaplan and his colleagues in the United States [12], they focused on the realistic heterogeneity in household income and consumption to examine the effect of the COVID-19 pandemic. Similarly, Bakar et al., and Correia et al., disclosed the results about the economic fallout due to the COVID-19 pandemic [16,17].

The effects of the COVID-19 pandemic on consumption and employment have also been considered in very recent studies [11,13,18]. However, a massive literature is available on food security. For instance, the determinants of food insecurity have been examined in various countries, including Ethiopia, Ghana, Zimbabwe, Kenya, Brazil, and Nigeria [19,20,21,22,23,24]. Likewise, studies on food security determinants have been conducted in Pakistan [25,26,27,28,29,30], but these studies have not examined the impact of COVID-19 on food insecurity.

A few studies discuss the impact of COVID-19 on food security. For instance, Laborde et al. investigated the impact of COVID-19 on food security through media tools and revealed that the lockdowns and mobility restrictions imposed brought consequences through the subsequent loss of income and purchasing power on food security [31], and O’Hara and Toussaint analyzed the crises of food access and the impact of COVID-19 on food security in Washington D.C., United States, and found that imposed social isolation exacerbated sociopolitical barriers and made conventional food solutions inadequate [32]. A study about the impact of COVID-19 on food security has been conducted in Africa too [33]. It is also revealed that lower income caused less access to food, and the food-insecure population eats less food and even remained hungry [34]. They also made changes in their consumption behavior [35,36].

In view of the literature, the research has not investigated the impact of COVID-19 on food insecurity in Pakistan so far. Hence, this study fills the research gap, and describes the answers to the following questions; (i) what is the impact of the COVID-19 pandemic on food insecurity, and (ii) its determinants and the household response to mitigate the food insecurity during the COVID-19 pandemic. The study’s strengths include its early administration, population-based assessment, and addressing multiple dimensions of food security. We implemented this survey at the beginning of a lockdown. As such, many respondents are likely experiencing job loss or disruption in daily activities. 

### Conceptual Framework

According to the report of Food and Agriculture Organization (FAO), people already struggling with hunger, health, and poverty are the most perilous during the COVID-19 pandemic [37,38]. Policy responses against COVID-19 in terms of quarantine, social distancing, traveling, and transportation restrictions have caused unemployment and, consequently, households’ incomes reduction. Households’ access to food is associated with their income level and available resources [18,39,40]. A reduction in the income of marginalized people affects their ability to purchase food [41]. Low-income people, already consuming low amounts of food, reduce their consumption even more, aggravating food security [42]. In addition, the epidemics such as MERS, HIV, AIDS, and Ebola have had a larger impact on marginalized and vulnerable women, children, the elderly, and the poor population [6,37].

Reductions in income and increased food prices urge poor households to adopt specific coping strategies to mitigate adverse effects of pandemics. Coping strategies and financial assistance improve the household ability to buy food items and thus reduce food insecurity. A conceptual framework through which COVID-19 has affected people is presented in Figure S1.

## 2. Materials and Methods

### 2.1. Data and Sampling

The Punjab province in Pakistan was taken as a case study for this research, as it is the most populated province of the country. [43]. Rural areas have a high proportion of the population [44]; as such, this province is more vulnerable to COVID-19. 

#### Sample Selection

The target population of this study is the people of Punjab, Pakistan. The sample size of this study was determined using Cochran’s [45] formula for large populations. The following equation represents the Cochran formula:

(1) $$N=Z^{2}pq/e^{2}$$

where *N* represents sample size; *Z* is the abscissa of normal curve that cuts off an area α at the tail; *e* is the desired level of precision; *p* is the estimated proportion of an attribute; *q* = 1 − *p* with the assumption of *p* = 0.5 (maximum variability) as the desired confidence interval, a ±5% precision level, and value of 1.96 for *Z*.

(2) $$\begin{array}{c} N={\left(1.96\right)}^{2}0.5\left(0.5\right)/{\left(0.051\right)}^{2}\\ N=0.9604/0.002601\\ N\approx 370 \end{array}$$

The sample size of this study was estimated to be 370. This non-probability sampling technique used in our study was also used in the prior studies i.e., [43,46] to collect data through an online survey from Punjab, Pakistan. Data collection from the respondents was the biggest challenge due to the COVID-19 pandemic, where “stay at home and practice social distancing” and other precautionary measures were implemented to prevent the outbreaks. Internet-based data were collected from Punjab with a snowball sampling technique, a non-probability sampling technique with specific advantages and disadvantages. With this technique, researchers can save time and costs and easily reach to the targeted populations in a specific study area. However, researchers have little control regarding oversampling, which may lead to sample bias. The reason for selecting this technique was the physical non-availability of respondents due to the COVID-19 pandemic and the budget constraints. It is very problematic to meet households one-by-one as the COVID-19 virus transmits from person to person [43,47]; people from around the world are converted on the internet, and it is difficult to target the desired population with other sampling techniques. For example, random sampling has randomness while selecting respondents. In quota, convenience, volunteer, and purposive sampling, respondents can refuse to participate in the study. In sequential sampling, sampling is taken at a given time interval and features a complex sampling design, where the basic sample size is not fixed in advance. The snowball method is best used for studying the impacts of diseases or pandemics while they are still present in the population [48].

The questionnaire was translated into the Urdu language for the better understanding of the respondents, and it was implemented using Google Sheets for Internet-based data collection and shared on social media platforms using Facebook and WhatsApp to obtain responses. Data were collected from May to September 2020 during the first wave of the COVID-19 pandemic in the country. The inclusion criterion in the study was an age of 17 years or above. Respondents’ responses were received from different districts of the province. (see Figure S2).

All respondents were informed about the purpose of the data collection and that their data would only be used for education and research purposes, which was mentioned in the questionnaire collected online. Participants were also informed that their data would be kept secure and safe. The questionnaire that was used is annexed in the Supplementary Materials section. The experimental design was presented in the committee meeting where it was confirmed and approved by the committee members.

### 2.2. Econometric Models

The household food insecurity access scale (HFIAS) model was applied to measure the level of food insecurity. Moreover, a logit regression model was used to analyze the determinants of food insecurity. This study used a coping strategy index to calculate households’ managing strategies. The details of the econometric models are described below.

#### 2.2.1. Household Food Insecurity Access Scale (HFIAS) Model

The HFIAS model was applied to estimate the food insecurity among households before and during the COVID-19 pandemic. The HFIAS model is a questionnaire that measures experienced food insecurity [49], and it has been widely used in earlier research for data collection, such as [50,51,52,53]. In Table 1, if a respondent responds “yes” to questions 1 to 3, then the respondent is experiencing “little hunger,” and if not, then “no hunger.” If a respondent answers “yes” for questions 4 to 5, then “moderate hunger,” and if a respondent replies “yes” to questions 6 to 8, then it shows “severe hunger”. The total HFIAS score can range from 1 to 8 in this study, indicating the degree of insecure food access. This technique was utilized in the previous research [54]. Table 1 presents the HFIAS questionnaire and the measurement method in detail. 

#### 2.2.2. Logit Regression Model

Various factors can influence household food insecurity levels. Herein, household food insecurity (dependent variable) was characterized; if a household has experienced food insecurity (FIS), then the value is 1; otherwise 0. The logit regression model fits the data to determine the factors that influence food insecurity at the household level. As stated in an earlier study, examining the relationship between food-insecure and food-secure households requires the use of discrete choice models, and hence, a logit regression model is a suitable. Logit model is used commonly when modeling a binary classification. Further, this model is simple to interpret and is extensively used in decision studies [55,56,57]. In our study we also used a binary dependent variable. Hence, this study followed the logit model for food insecurity applied by Cameron et al. [58] as the following:

(3) $$\mathrm{Pr}(\mathrm{Yji}=1/X)=ø(x^{\prime }\beta )=\frac{{\mathrm{ex}}^{\prime }\beta }{1+{\mathrm{ex}}^{\prime }\beta }$$

where ø(.) is the cumulative distribution function (CDF). The model is estimated using maximum likelihood estimation (MLE), which assumes independence across observations and that the maximum likelihood (ML) estimator β is consistent and asymptotically normally distributed. Estimations rely on the assumption that the latent error term is normally distributed and homoscedastic and is written as follows:

Y = β_0_ + β_1_X_1_ + β_2_X_2_ + β_3_X_3_ + ………. + β_n_X_n_ + ε (4)

where Y represents the food security status (1 if the household is food insecure and 0 otherwise);

X_1_ = gender of the household head (1 if male; 0 for female);

X_2_ = age of the household head (number of years);

X_3_ = education of the household head (school years);

X_4_ = marital status (0 if single; 1 if married);

X_5_ = family size (total number of family members in a household);

X_6_ = professional category (1 if daily wage worker; 2 if government employee; 3 if business; 4 if laboror);

X_7_ = household savings in PKR;

X_8_ = income category (1 if income less than or equal to 17,000 PKR (minimum wage rate); 2 if income higher than 17,000 PKR);

X_9_ = quarantine (1 if the household has quarantined, 0 otherwise);

X_10_ = financial support or aid (1 if received financial support during the COVID-19 period; otherwise 0);

X_11_ = health insurance (1 if respondent has health insurance; otherwise 0);

X_12_ = community size (1 if large community size; 0 otherwise);

X_13_ = location (0 if respondents belongs to rural; 1 if urban);

ε = an error term.

To analyze the data for obtaining results, the STATA 13 software package was used. To measure coping strategies, a household food security coping index was formed, which consisted of a series of questions based on how households managed the reduction of food consumption. The results are described with a simple numeric score to follow the previous research [40,59].

#### 2.2.3. Coping Strategy Index

Households adopt several coping strategies, and, hence, food insecurity can be measured using a coping strategy index (CSI); a previous study also used this technique [59]. This method measures the behavioral changes made at the household level in adjusting to food insecurity, while the frequency of a household’s coping strategies addresses a shortage in food supply and is a rapid assessment of a household’s current food security. A higher score in the CSI indicates greater food insecurity. This study examined a total of 12 coping strategies, aggregated into four basic groups: (1) Dietary change, e.g., eating cheaper food; (2) increasing access to the short-term food supply, e.g., borrowing or asking others for help; (3) decreasing the number of people to feed, e.g., sending family members elsewhere; (4) rationing food, e.g., family members skipping meals or prioritizing feeding kids. These four basic food coping strategies were aggregated and weighted into an index that summarized the indicators of food insecurity. The individual responses were recorded into five Likert scales, and each category was weighted, where 1 = no adopted category, 2 = one time per week adopted, 3 = 1–2 times per week adopted, 4 = 3–6 times/week adopted, and 5 = adopted all the time. Relative weights were assigned to each category depending on the level of severity. A high score of “5” indicates a more severe category, while “4” indicates relatively lower severity. The relative weights were multiplied with the individual responses. Finally, scores for the relative frequency of how often households relied on different coping strategies were assigned, as suggested by Maxwell in his two different studies [40,59] (see Table A1).

## 3. Results

### 3.1. Household Characteristics

Table 2 illustrates a detailed summary of the household statistics. It was revealed that the households were highly concerned about the infectious disease, and almost 5.1% of the households reported that their family members were infected with the COVID-19 pandemic, while 1.1% reported that their family members had died due to this disease. Moreover, the households revealed difficulty in accessing essential supplies for living. The households showed medium-to-high difficulty accessing essential goods and supplies due to government policies implemented in response to COVID-19, representing 83.7% of the respondents. The study also gave due coverage to female households heads, and 14.9% of the total responses were received from women. Furthermore, the responses to the survey received from rural respondents represented 40.4% of the total, while urban respondents involved 55.3%. The percentages of the households regarding gender (male and female), location (rural and urban), and marital status (single or married) are shown in Table A2.

This section presents detailed results for the HFIAS model, logit regression, and coping strategy index models.

### 3.2. Results for the Household Food Insecurity Access Scale

Due to the COVID-19 pandemic, food insecurity level worsened and increased significantly, while the households reported relatively better food security in the pre-COVID-19 pandemic; Table 3 summarizes the food insecurity access scale for a different level of food severity prior to and during the COVID-19 pandemic, such as severe hunger, moderate hunger, and no or little hunger. The results revealed that severe hunger level increased among 44.2% of the households from 17.4% pre-COVID-19 to 61.6% to during COVID-19, while the moderate hunger level increased among 18.74% households due to the COVID-19 pandemic from pre 23.09% to 41.83% during COVID-19. However, the no or little hunger level increased among only 2.2% of the households which is 10.8% pre COVID and 13% during COVID-19. 

### 3.3. Determinants of Food Insecurity during COVID-19—Logit Regression Model

The present study focused on determining factors of food security, and the results are shown in Table 4. Further details of these results are described in the following sections.

#### 3.3.1. Role of Demographic Variables

The results of the logit regression model, in Table 4, reveal that the coefficients of age and gender are negatively significant. It shows that female-headed families found higher food insecurity than male-headed families. The coefficients of education and marital status have negative values but a non-significant association with food insecurity. However, family size has a positive and statistically significant coefficient, and it indicates that the households with more family members experienced more food insecurity. Mango and their colleagues [21] conducted a study in Zimbabwe and revealed that age, education, and the amount of labor by the household head were found to influence household food security positively. Similarly, some other studies also found that household food security is considerably affected by the household head’s education level, age, gender, family size, income, and family structure [19,21,22,30,60]. 

#### 3.3.2. Role of Location and Community Size

This study shows that the household location variable was found to be positively and statistically significant with food insecurity, which denotes that people living in urban areas experienced higher food insecurity than their counterparts during the COVID-19 pandemic. However, a negative and statistically non-significant association of community size with food insecurity was found, which shows that the households in less populated communities experienced less food insecurity. Community volunteers provide food to overcome the food insecurity situation during the pandemic in congested communities, but maybe the services and provided food are not sufficient to them. The available food has been found to be more expensive in urban areas thus leading to higher food insecurity in urban areas (see Table 4). These findings are in line with the previous study [61]. 

#### 3.3.3. Role of Social Distancing Policies

This study revealed that the quarantine variable has a positive coefficient value and is statistically significant. It shows that households who adopted precautionary measures or remained in quarantine during the COVID-19 period faced higher food insecurity. (Table 4). Social distancing and quarantine policies are essential to avoid infections, and health is significantly influenced by wealth. Health conditions are fundamental in terms of food insecurity [61].

#### 3.3.4. Role of Professions and Income Categories

The household professions of laborers, government employees, business owners, and landlords had a non-significant association with food insecurity (Table 4). In addition, the households in higher-income groups were negatively and non-significantly associated with food insecurity. In contrast, low-income households face food insecurity, as food-insecure people do not have sufficient money to meet their dietary requirements [25]. Unemployed people do not have enough money to purchase food items. Activities that generate income can reduce the poverty and, ultimately, result in a reduction in food insecurity [20].

#### 3.3.5. Role of Savings, Financial Aid, and Health Insurance

This study revealed that health insurance and saving rates during the COVID-19 pandemic were non-significantly associated with the food insecurity (see Table 4). Moreover, the financial aid obtained during the pandemic was negatively significantly associated with food insecurity. It shows that the households that received income support or aid are less food insecure than their counterparts. The results of a previous study is in line with our study results and showed that among those infected by the disease, low-income households spend a major part of their income on health-related issues, and the money left to buy food is not sufficient, thereby causing food insecurity [61].

### 3.4. Purchasing Power Shock

The impact of COVID-19 on household income was assessed by asking the following questions from the respondents: (1) Please indicate the “monthly loss of income” (in Pakistan rupees from all sources) due to COVID-19; (2) please reveal the perceived “loss of employment” due to COVID-19 on a scale of the point from 1 to 10; (3) please detail the perceived “increase in monthly household debt” due to COVID-19 on a point scale from 1 to 10; (4) please describe the perceived “increase in food prices” due to COVID-19 on a point scale from 1 to 10.

Based on respondents’ answers, results divulge that more than 18% of the sample households lost a monthly income around 15,000 PKR, while more than 72% of the sample households reported income loss less than 10,000 PKR/month. More than 80% households faced a higher employment loss due to the COVID-19 pandemic. Additionally, 91% of sampled households indicated that food prices increased by more than 5 to 10. Further, 83% of the sampled households reported that their household debts increased by 5–10 points on the Likert scale due to the COVID-19 pandemic, and hence household purchasing power reduced significantly because of the COVID-19 pandemic (see Table 5).

### 3.5. Household Coping Strategies

Coping strategy index data were calculated to rank household coping measures. The maximum score obtained by the basic strategy adopted in response to food insecurity was rationing strategies, as adopted by 63.9% of the households, which indicates that the prevalence of food insecurity was highest in this 63.9% of the households. Moreover, the basic strategies of “decrease in the number of people” and “increasing short-term food availability” were adopted by 85.6% and 79.6% of the households, respectively; however, the “dietary change” basic coping strategy was adopted by 97% of the sampled households, indicating that during the COVID-19 pandemic, the households experienced food insecurity as a result of changing their diet (see Table 6).

### 3.6. Financial Support/Aid

The households were provided with financial support by different agencies, such as government organizations and philanthropists, to mitigate adverse income shocks. To assess the impact of financial support or aid, the following questions were asked: (1) Did you receive any government support or aid from any institution? (2) Do you feel that government aid or financial support from any other source helped you meet your expenses? (3) Do you feel that this aid improved your ability to fulfill your food expenses? (4) Where did you get this aid from? (5) What percentage of the aid you received has been consumed? (6) Do you have health insurance?

In the sampled households, 79.7% did not possess health insurance. Only 5.7% of the respondents reported health insurance, while 14.6% did not answer this question. The reason for the lack of health insurance may be because the provincial government provides free medical services to citizens in public hospitals. In addition, 10.8% of the households received financial support or other assistance; hence, this assistance helped the households to meet their basic food expenses (8.4% of households) and raised their ability to buy food items (14.9% of households). The major financial supporters were (1) government organizations (contributed 7.9%), (2) community groups (contributed 3.5%), (3) friends and relatives (contributed 5.1%), (4) private charity organizations (contributed 5.4%), and (5) workplace contributions (contributed 2.7%) among the sampled households (see Table A3). This assistance was provided during peak days of the first wave of the COVID-19 pandemic in the province. Consequently, financial support helped marginalized households to improve their food security during the COVID-19 pandemic period.

## 4. Discussion

Our study aims to assess the impact of COVID-19 on food insecurity in Punjab, a province of Pakistan, its related determinants, and people’s response to mitigate adverse income shocks.

The results found that severe food insecurity increased by 44.2% in households due to the COVID-19 pandemic. This rise in food insecurity presents many potential health impacts. Many researchers, including Bakalis et al. [62], Cook et al. [63], Gundersen and Ziliak [64], and Aguero et al. [65], associate undernourishment with adverse health outcomes, such as chronic conditions, mental health challenges, and increased risk of mortality. This finding is an indication of the poor physical and health conditions among the people in the study area. Adverse health outcomes that stem from food insecurity are of direct importance to healthcare professionals and the policymakers and program administrators charged with improving health and well-being. Hamelin et al. [66] identified three potential consequences of food insecurity, i.e., physical, psychological, and socio-familial. Physical manifestation could be reflected into lack of concentration and low work capacity. Psychological manifestations relate to lack of access to food, creating enormous stress. The socio-familial factor covers modifications of eating patterns, and disrupted household dynamics of food acquisition and management. Gundersen and Ziliak [64] found that food-insecure children are at least twice as likely to report being in poor health; food-insecure teenagers have limitations in activities of daily living comparable to those of food-secure teenagers.

McIntyre et al., [67] found depression or suicide among youth ages 14–25 years in households experiencing hunger were 2–3 times higher than among youth in households without hunger. Cook et al. [63] found odds of poor health among children in households with food insecurity were 2.14 times higher than among children in food-secure households. Park et al. [68] revealed that iron deficiency among pregnant women was 2.90 times higher due to food insecurity than their counterparts. Seligman et al. [69] found that food-insecure individuals have approximately twice the odds of experiencing diabetes, compared to food-secure individuals. They further found that food insecurity is associated with a 20% increase in the risk of hypertension and a 30% increase in the risk of hyperlipidemia.

The finding of Punjab, Pakistan supports the expectations of the FAO [70,71], the WFP [72], and the WTO, as well as the projections of many scholars [73,74], who say that the spread of COVID-19 may bring damage to food security, particularly painful in the least developed and developing economies.

The results of the logit regression model demonstrated that age has a negative impact on food insecurity. Our study results are supported by a previous study, as Hofferth [75] revealed that older people are more mature and may have better experiences obtaining the resources they require. In contrast, a study analyzed by Khan et al. [76] reveals that work efficiency decreases as age increases and increases the chances of food insecurity.

The results of our study documented a negative and statistically significant coefficient of gender. This shows that female-headed families experienced higher food insecurity than male-headed families. Maharajan and Joshi [77] argued that the husband’s death, separation, and husband’s migration outside the city or village may result in food insecurity among female-heading households. These households possess less physical access for agricultural activities, possess less livestock, and cultivate the land they own, etc. This makes these households more likely to be food insecure. In contrast, another argument is that food activities (purchasing, preparation, etc.) are concerned with females, so a household having a female household head is more independent regarding spending on food than a household headed by a male. Thus, in this case, a household having a female head is less likely to be food insecure.

Results of this study found that family size has a positive coefficient. Additionally, an increase in family size tends to exert more pressure on consumption in the household. The larger the household, the higher the chances of being food insecure, as it requires more money to meet both food and other daily needs for more persons. Our present study documented that the quarantine coefficient is positive and statistically significantly associated with food insecurity. The studies by Kodish et al. [78] and Wernery and Woo [79] found movement restriction policies and quarantines introduced during MERS, Ebola, and other more local outbreaks to affect the food industry distribution and retailing of many staples food substantially, resulting in food insecurity. When affordability and availability of alternative protein sources are deteriorated by economic factors, local outbreaks of other diseases may substantially aggravate both the health and food security status of broad segments of the population.

It is found in our study that financial support is negatively associated with food insecurity. In particular, direct financial support for the emergency food needs, long term food provision to families, and expanding food assistance is urgently needed. It is critical for Punjab, Pakistan to expand the “Ehsas Emergency Cash Program” to support low-income food-insecure families of the province.

Further, results demonstrate that household purchasing power reduced significantly due to the COVID-19 pandemic because of income shocks in a higher loss of income, greater unemployment, increased food prices, and high debts, increasing poverty and making food access strenuous. According to FAO’s food security report, a key reason for the growing food insecurity in developing countries is that many people cannot afford the increasing cost of healthy diets. At the same time, the nutritional status of vulnerable population groups has been deteriorated due to the economic impacts of COVID-19 [71]. This correlates with FAO’s estimation that the cost of a healthy diet in 2020 has exceeded the international poverty line, making it unaffordable for the poor and thus fueling food insecurity in most developing countries, particularly in Sub-Saharan Africa and Southern Asia [71]. The UNCTAD [80] also acknowledged the countries of Sub-Saharan Africa to be particularly exposed to demand-side risks of food access during the COVID-19 crisis, including contracting incomes, downturns in economic growth, undernutrition, and micronutrient deficiencies in response to income shocks.

Our study results show that the household, in response to the increasing food insecurity, has changed dietary habits, led to rationing food, decreased the number of people, and increased short-term food availability. Niles et al. [81] found that lower economic access to food forced many food-insecure households to disrupt eating, cut meals, eat less to stretch their food, or even go hungry. Bakalis et al. [62], and Poudel et al. [35], and Siche [36,82] witnessed significant adverse effects of SARS, MERS, avian and swine flu, Ebola, and other outbreaks on food consumption behavior.

## 5. Conclusions

The population facing food insecurity has been hit more adversely by the far-reaching impact of the COVID-19 pandemic. The impact of the pandemic mainly derives from lockdown and mobility restrictions imposed by governments and the consequences that the subsequent loss of income and less purchasing power has on food security. The food insecurity among households has increased 44.2% due to the COVID-19 pandemic. The households with more family members, those that remained in quarantine, and low-income families experienced more food insecurity. In response to the income shocks, households adopted several coping strategies, such as rationing, dietary changes, less eating, and increased short-term food availability. Among the households who received income support or aid from government and charity organizations, they found less food insecurity than their counterparts. The financial support and aids helped marginalized groups to improve their food security during the COVID-19 pandemic.

### 5.1. Policy Recommendations 

In view of the results, the following suggestions have been provided.

- The government should ensure food availability at lower prices to enable access for poor populations.
- People’s income-raising activities should be protected by ensuring smooth economic flow by applying smart lockdown (smart lockdown means if the area has higher confirmed cases of the COVID-19 pandemic disease, that area should be under lockdown, but the areas with a low positivity rate would not be imposed with a lockdown.).
- The prevalence of food insecurity in poor families was higher; therefore, the government and stakeholders should provide more financial assistance to poor families.
- Programs similar to the Ehsas income program (the Ehsas Program is the program which supports to low-income families by providing financial assistance by the government of Pakistan) should expand to support the affected population.
- In the food dimensions, physical and economic access must be considered. This research demonstrates the need to increase food assistance programs and provide resources to remove food access barriers now and likely in the future during public health emergencies.
- In the short run, some targeted interventions such as cash transfer or subsidies are helpful, but in the long run, a better solution is to have economic growth, which ensures not only an increase in income but also help in making it possible to provide ample opportunities to the poor people to gain access to food, health, and jobs.

#### Limitation of the Study

Although this study covered various aspects about food insecurity, it still has the following limitations.

Data were collected through online sources due to the COVID-19 lockdown. Though our respondent population was internet users, this was a convenience sample; further research expands similar questions with representative samples across other provinces and populations. Future research may reduce the sample bias, and data can be collected from those households who do not have internet access and/or a smart phone. It can also be examined the evolution of the food-security impact, and how various interventions, including food assistance and healthcare screenings, may affect food insecurity outcomes as COVID-19 unfolds.

## Figures and Tables

**Table 1:** Measurement scales of the household food insecurity access scale (HFIAS) model.

Sr.	Scale	Household Questions	Intended Meaning with Respect to Food Adequacy If Responding with “Yes.”	Assumed Severity of Food Insecurity
1	Uncertainty and worry about food	Did you worry that the household lacks food?	Worried about how to procure food	Little or no hunger
2	Unable to eat preferred food	Did anyone from the household eat food which you did not prefer?	Food preference and compromising quality	Little or no hunger
3	Consumption of few kinds of food	Did anyone from the household eat a limited variety of food?	Food varieties compromise quality	Little or no hunger
4	Unable to eat healthy and nutritious food	Were you unable to cook healthy and nutritious meals?	Healthy and nutritious food	Moderate hunger
5	Eating smaller meals	Did anyone from the household eat fewer meals due to a lack of food?	Food preferences of children	Moderate hunger
6	Eating fewer meals in a day	Have you gone a whole day without eating/eating fewer meals?	Skipping meals	Severe hunger
7	No food in the house	Do you unable to cook any type of food due to not availbe?	Running out of food completely	Severe hunger
8	Run out of food	Would you go hunting for wild food?	Experiencing hunger at the household level	Severe hunger

**Table 2:** Summary statistics.

Household Characteristics	Mean	Standard Deviation (SD)
Age of head	29	9.06
Education of head	12	4.02
Income level of head (PKR)	39,840	48,828
	Frequency	Percentage
Reason head is concerned about COVID-19		
Family member infected	19	5.1
Family member died	4	1.1
Head’s gender		
Male	300	81.3
Female	55	14.9
Head’s marital status		
Single	162	43.9
Married	192	52
Location		
Rural	149	40.4
Urban	204	55.3
Head’s profession	144	39.0
Labourer	102	27.7
Daily wage and private worker		
Government job	49	13.3
Own business and Landowner	55	14.9
Community size		
Small	317	215.5
Large	36	9.8
Difficulty in accessing essential supplies		
No	1	0.3
Low	36	9.8
Medium and High	309	83.7
Sample size (*n*) = 370		

**Table 3:** The impact of COVID-19 on food insecurity.

Food Insecurity	Scale	Pre-COVID-19 (%Age Households)	During-COVID-19 (%Age Households)	Impact of COVID-19 (%Age Households)
Severe hunger	Insufficient food intake and resulting physical consequences	17.4	61.6	44.2
Moderate hunger	Insufficient quality (includes variety and preferences of the type of food)	23.09	41.83	18.74
Little or no hunger	Anxiety and uncertainty about the household food supply	10.8	13	2.2
	Sample size (*n*)	370		

**Table 4:** Results of the logit regression model.

Variables	Coefficients	Standard Error	T	*p* > |t|
Age	−0.0071 *	0.0034	−2.08	0.038
Education	−0.0070	0.0062	−1.12	0.262
Location (rural/urban)	0.0678 ***	0.0390	1.74	0.083
Family size/members	0.0407 *	0.0144	2.83	0.005
Gender	−0.1482 *	0.0510	−2.90	0.004
Marital status	−0.0701	0.0573	−1.22	0.222
Quarantine	0.1946 *	0.0668	2.91	0.004
Health insurance	0.1067	0.0681	1.57	0.118
Saving	0.00000139	0.00000189	0.74	0.462
Community size	−0.0285	0.0538	−0.53	0.596
Financial support/aid	−0.2159 *	0.0561	−3.85	0.000
Profession category				
2 (government jobs)	−0.1011	0.0911	−1.11	0.268
3 (businesses)	−0.0529	0.0425	−1.24	0.214
4 (laborers)	−0.0227	0.0494	−0.46	0.645
Income category				
Low	0.0643 *	0.0751	0.86	0.092
High	−0.0667	0.1458	−0.46	0.647
Constant	2.1782 *	0.1749	12.45	0.000
Adjusted *R*^2^ Root-mean-square error Probability > *F*	0.2264 0.34377 0.000	Sample size (*n*) *F* (16, 351)	370 7.71	

* and *** represent levels of significance (α) at 10% and 1%, respectively.

**Table 5:** Purchasing power shock.

Description		Frequency	Percentage (%)
Income loss	No impact at all	12	4.1
	Low impact (income loss <10,000)	215	72.8
	Medium impact (income loss of 10,000 to 15,000)	13	4.5
	High impact (income loss >15,000)	55	18.6
Employment loss	Less affected <2	28	7.9
	Moderately affected (2–5)	33	9.3
	Highly affected (5–10)	293	82.8
Household debt	No affect	23	6.7
	Less than 5	35	17
	More affected (5–10)	284	83
Food price rise	No rise	5	1.4
	Less than 5	26	7.6
	More affected (5–10)	315	91
Sample size (*n*) = 370			

**Table 6:** Households’ coping strategy index scores.

Sr #	Coping Strategy	Coping Strategy Index Score	Household Percentage (%)
1	Dietary change	939	97
2	Increase in short-term household food availability	3765	79.6
3	Decrease in the number of people to feed	2504	85.6
4	Rationing	7804	63.9

## Data Availability

Data are not publicly available due to the confidentiality of the respondents.

## Supplementary Materials

The following are available online at https://www.mdpi.com/article/10.3390/healthcare9060621/s1, Figure S1: Conceptual framework, Figure S2: Study Area.

## Appendix A

**Table A1:** Construction of the coping strategy index model.

Sr. #	Coping Strategy	Basic Category	Relative Weight	Everyday	3–6 times/week	1–2 times/week	<1 time/week	Never	Total Score
1	Less preferred	Dietary change	3						
2	Borrowed	Increase in short-term availability	3						
3	Fewer purchases with credit		3						
4	Wild food		3						
5	Eating seed stock		3						
6	Household members sent elsewhere	Decrease in number of people to feed	4						
7	Begging for food		4						
8	Limit portion on food	Rationing	4						
9	Restricted adult intake		3						
10	Feed workers		3						
11	Reduced meals		4						
12	Skip days		4						
Total index score									

**Table A2:** Sampling percentage distribution.

Characteristics	Population (%)	Sample (%)
Gender		
Male	50.80	81.3
Female	49.20	14.9
Rural and urban population		
Rural	63.90	40.4
Urban	35.10	55.3
Marital status		
Single	31.84	43.9
Married	61.76	52
Divorced	0.45	0.3

Sources include the authors’ calculations and [43,44].

**Table A3:** Health insurance and financial assistance.

Descriptions	Response	Frequency	Percent (%)
Household has health insurance	No	294	79.7
	Yes	21	5.7
Received aid	No	315	85.4
	Yes	40	10.8
Aid helped in mitigating expenditure	No	285	77.2
	Yes	31	8.4
Aid helped in raising the ability to buy food items	No	264	71.5
	Yes	55	14.9
Aid was received from organizations	(a) Community	13	3.5
	(b) Friends/family	19	5.1
	(c) Government department	29	7.9
	(d) Other places	183	49.6
	(e) Private charity organization	20	5.4
	(f) Workplace	10	2.7
Percentage of aid used during COVID-19	Equal to 20%	41	11.1
	Equal to 40%	53	32.7
	Equal 60%	68	18.5
	Equal to 80%	48	13
	Equal to 100%	27	7.3
