# Supplementary figures and images for: COVID-19 Pandemic, Determinants of Food Insecurity, and Household Mitigation Measures: A Case Study of Punjab, Pakistan
Source: Healthcare (Basel). 2021 May 22;9(6):621. doi: 10.3390/healthcare9060621 (PMC8224546; doi:10.3390/healthcare9060621)

## SUPPLEMENTRY MATERIAL

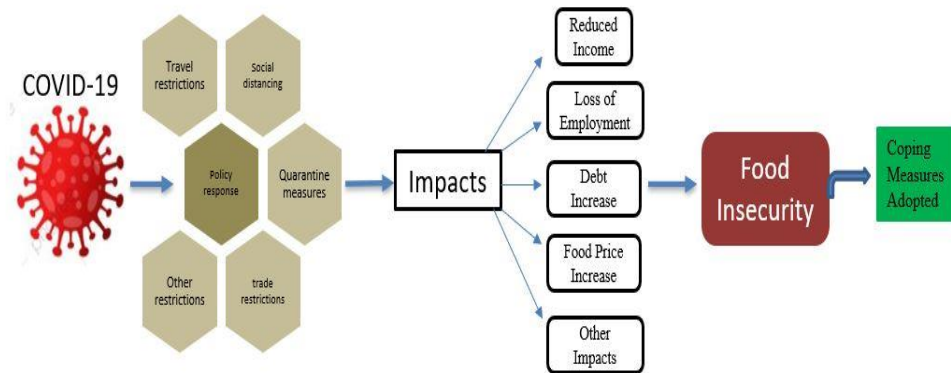

Figure S1. Conceptual framework

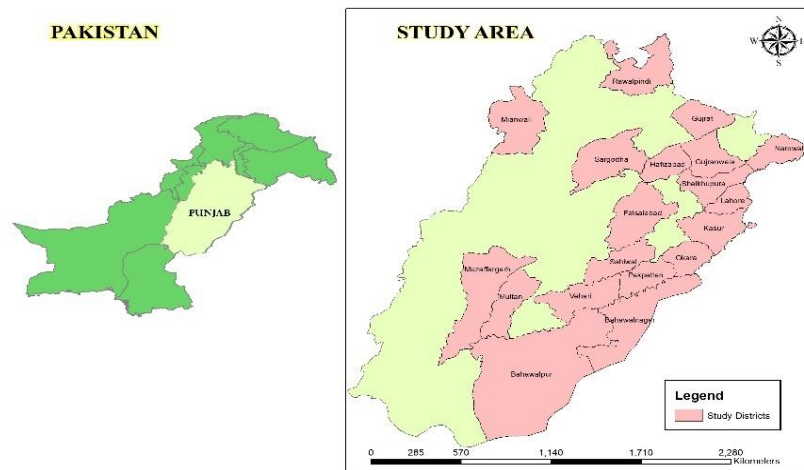

Figure S2. Study area.

Supplement: Supplementary file 1 [file healthcare-09-00621-s001.zip › healthcare-1173228-supplementary.pdf]
